# Reduced CD146 expression promotes tumorigenesis and cancer stemness in colorectal cancer through activating Wnt/β-catenin signaling

**DOI:** 10.18632/oncotarget.9930

**Published:** 2016-06-09

**Authors:** Dan Liu, Lei Du, Dong Chen, Zhongde Ye, Hongxia Duan, Tao Tu, Jing Feng, Yili Yang, Quan Chen, Xiyun Yan

**Affiliations:** ^1^ Key Laboratory of Protein and Peptide Pharmaceuticals, Institute of Biophysics, Chinese Academy of Sciences, Beijing 100101, China; ^2^ State Key Laboratory of Biomembrane and Membrane Biotechnology, Institute of Zoology, Chinese Academy of Sciences, Beijing 100101, China; ^3^ Department of Pathology, Beijing Anzhen Hospital, Capital Medical University, Beijing 100029, China; ^4^ Department of Colorectal Surgery, Xinhua Hospital, Shanghai Jiaotong University School of Medicine, Shanghai 200092, China; ^5^ College of Life Sciences, University of Chinese Academy of Sciences, Beijing 100049, China

**Keywords:** stemness, CD146, tumorigenesis, Wnt/β-catenin, colorectal cancer

## Abstract

Cancer stemness drives tumor progression and drug resistance, representing a challenge to cancer eradication. Compelling evidence indicates that cancer cells can reenter the stem cell state due to the reprogramming of self-renewal machinery. Here, we show that CD146 knockdown induces stem cell properties in colorectal cancer (CRC) cells through activating canonical Wnt signaling. shRNA-mediated CD146 knockdown in CRC cells facilitates tumor initiation in serial xenotransplantation experiments. Moreover, upon CD146 knockdown, CRC cells show elevated expression of specific cancer stem cell (CSC) markers, increased sphere and clone formation as well as drug resistance *in vitro*. Mechanistically, our findings provide evidence that CD146 expression negatively correlates with canonical Wnt/β-catenin activity in CRC cell lines and primary CRC specimens. Knockdown of CD146 results in inhibition of NF-κB/p65-initiated GSK-3β expression, subsequently promoting nuclear translocation and activation of β-catenin, and as a consequence restoring stem cell phenotypes in differentiated CRC cells. Together, our data strongly suggest that CD146 functions as a suppressor of tumorigenesis and cancer stemness in CRC through inactivating the canonical Wnt/β-catenin cascade. Our findings provide important insights into stem cell plasticity and the multifunctional role of CD146 in CRC progression.

## INTRODUCTION

Cancer is one of the major health concerns to humans, with colorectal cancer (CRC) being the third-leading cause of cancer deaths in recent years [1, 2]. A large body of basic research and clinical evidence has established that cancer stemness contributes to carcinogenesis, tumor relapse and chemoresistance in traditional cancer therapeutics [3, 4]. Cancer stem cells (CSCs) have been identified and isolated from acute myeloid leukemia and various solid tumor types based on the expression of putative surface markers, such as CD44 and CD133 [5, 6]. However, with the evolution of the CSC model, newly emerging evidence has shaped our understanding of stemness and cancer cell plasticity [7–9]. Phenotypic transition and functional heterogeneity have been observed in cancer cells of different malignancies, including breast cancer [10], prostate cancer [11] and hepatocellular carcinoma [12]. In response to specific environmental stimuli, well-differentiated cancer cells can convert to a stem cell state characterized by self-renewal, differentiation and tumor-initiating potential. Thus, stemness is unlikely to be a rigid cellular state, but instead might be characterized by a set of more dynamic features influenced by the nature of the microenvironment. Acquisition of a stem cell-like phenotype in non-tumorigenic cancer cells may contribute to resistance towards therapy and subsequently to cancer relapse. Therefore, a more comprehensive view of cancer stemness should greatly assist with the development of CSC-targeted strategies and ultimately, the eradication of most, if not all, types of cancer. To date, however, the factors and regulatory mechanisms determining cell stemness, especially in colorectal cancer, are poorly understood.

Various extrinsic cues and intrinsic signaling pathways, such as Wnt, Notch and Hedgehog signals, are involved in maintenance of stemness [13]. The evolutionarily conserved Wnt signaling pathway is essential for embryonic development and cancer progression. Wnt signals can be classified into the non-canonical and canonical branches [14]. The non-canonical cascade is mediated by JNK or Ca^2+^ and is required for cell polarity in organogenesis and tumor invasion [15]. For the canonical Wnt pathway, β-catenin acts as a key downstream effector, which is typically phosphorylated by the destruction complex composed of adenomatous polyposis coli (APC), scaffolding protein Axin, casein kinase 1 (CK-1) and glycogen synthase kinase 3β (GSK-3β), and then degraded through the ubiquitination and proteasome system [16]. The binding of wnt ligands to the Frizzled receptors or constitutive mutations can lead to the stabilization and nuclear translocation of β-catenin, and subsequently binding with TCF/LEF transcription factors. This transactivation induces the up-regulation of specific Wnt/β-catenin target genes involved in cell proliferation and stem cell fate-determination [17]. More importantly, the canonical pathway is involved in the maintenance of intestinal homeostasis in mammals [18]. Deregulation of β-catenin can cause epithelial hyperplasia and malignant transformation towards colorectal carcinomas [19]. Furthermore, the Wnt/β-catenin signaling pathway facilitates the self-renewal of CSCs [20–22]. It has been reported that the aberrant activation of β-catenin could restore stem cell phenotypes in differentiated cancer cells and thus functionally defined colorectal CSCs [23]. These findings imply that the canonical Wnt signaling cascade also participates in establishing the phenotypic plasticity of cancer cells.

In addition to Wnt ligands, the melanoma adhesion molecule CD146 participates in Wnt signal transduction during embryonic development [24]. CD146 (also known as MCAM and MUC18), is a member of the immunoglobulin superfamily. It is required for cell adhesion, inflammation, vasculogenesis [25–28]. It is now established that CD146 facilitates malignant progression of solid tumors by promoting angiogenesis and metastasis [29–32]. A small number of studies also indicated that CD146 plays a potential role in tumorigenesis. It has been reported that CD146 increases tumorigenicity of melanoma SB-2 cells and prostate cancer cells LNCaP in nude mice [33, 34]. In malignant rhabdoid tumor, CD146 defines a tumorigenic subpopulation possessing high self-renewal potential [35]. Recently, it has been reported that CD146-positive cells in human sarcomas enrich in tumor-propagating cells [36]. In contrast, CD146 appears to act as a suppressor for carcinogenesis in breast cancer and certain mesenchymal neoplasms [37, 38]. Moreover, CD146 has been found to be lower in tumorigenic melanoma spheres compared with the corresponding adherent cells [39]. It is conceivable that these conflicting results reflect multifaceted effects of CD146 on tumor progression in a context-dependent manner. In addition, CD146 has also been identified as a pluripotent marker for mesenchymal stem cells (MSCs) [40, 41]. Based on these previous findings, we hypothesized that CD146 exerts potential effects on cancer cell stemness. Here, we demonstrated that CD146 functions as a negative regulator of stem cell properties in colorectal cancer through inhibiting the Wnt/β-catenin signaling pathway.

## RESULTS

### Reduced CD146 expression promotes tumorigenesis of CRC

**Figure 1:**
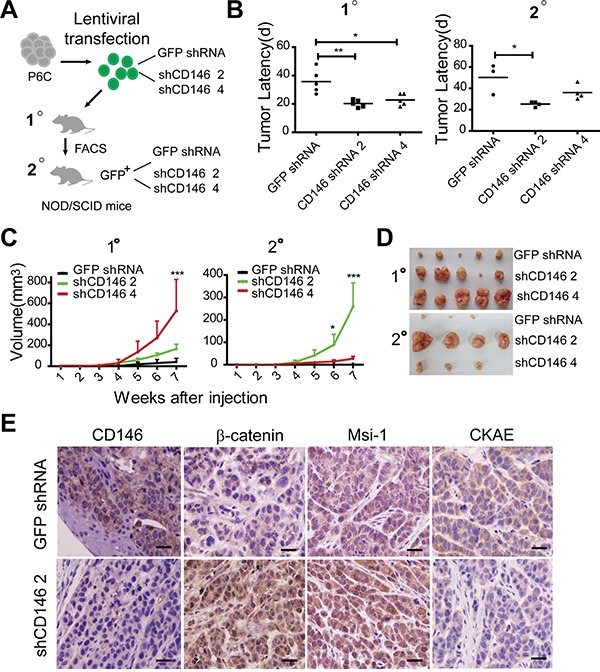
Reduced CD146 expression promotes tumorigenesis of CRC **A.** Experimental strategy. 100 P6C cells were used for each injection. **B.** Reduced CD146 expression accelerates tumor formation in serial transplantations. Tumor incidence and latency (timepoint at which tumors become visible) were monitored for three months post-transplantation. *P<0.05, **P<0.01. **C.** Tumor volumes and growth curves were measured in serial transplantations. Significance of differences at indicated time point were determined by two-way ANOVA analysis, *P<0.05, ***P<0.001. Error bars, mean ± s.d. **D.** Representation of human colorectal xenografts in serial transplantations. Only three of four secondary xenografts in shCD146 4 group are shown due to the accidental death of one mouse. **E.** Immunohistochemical staining of CD146, β-catenin, Msi-1 and pan-Cytokeratin (CKAE) in first xenografts. Scale represents 50 μm.

To evaluate the effects of CD146 on colorectal cancer stemness and tumorigenesis, we conducted serial xenograft transplantations in NOD/SCID mice (Figure 1A). P6C is a CD44^hi^ primary CRC cell line of tumor-initiating potential (Supplementary Figure S1) [42]. Furthermore, CD44^+^CD133^+^ cell population has been reported to enrich in colorectal tumor-initiating cells [43, 44]. Therefore, we also isolated CD44^+^CD133^+^ cell fraction from established human CRC cell line SW480 for parallel tests (to the primary cancer cell line P6C) (Supplementary Figure S2). Firstly, lentivirus-mediated shRNAs against CD146 were transfected into P6C and SW480 fraction cells (non-effective shRNA with GFP tag as a negative control). Knockdown efficiency of CD146 was verified by qRT-PCR in combination with flow cytometry (FACS) analysis (Supplementary Figure S3). One hundred single cells of stable monoclones selected from P6C-transfected groups (shCD146 2, shCD146 4 and GFP shRNA) were subcutaneously injected into NOD/SCID mice. In the first transplantations, we traced tumor formation for 7 weeks, until which time point both P6C cells in CD146 knockdown and control groups formed tumors in all five injected mice, while xenografts derived from CD146 knockdown cells developed significantly earlier than those of the control group (Figure 1B). In the secondary transplantations, cell fractions isolated from first xenografts in shCD146 2 and shCD146 4 group developed tumors in four out of five mice, whereas cells fraction derived from GFP shRNA group initiated tumors only in three of five mice. In addition, the tumor latency of secondary xenografts in shCD146 2 group was much shorter than that of the GFP shRNA group (Figure 1B). The differences observed in tumorigenesis imply that tumor-initiating efficiency negatively correlates with the expression levels of CD146. Moreover, we found that CD146 reduction facilitated the growth of xenografts in serial transplantations (Figure 1C and 1D). Similar results were observed in the serial transplantations of SW480 fraction (Supplementary Figure S4).

Furthermore, immunohistochemical analysis showed that the first xenografts in shCD146 2 group retained relative low expression level of CD146. In addition, the tumor cells in these xenografts exhibited strong staining for nuclear β-catenin and stem cell marker Musashi-1 (Msi-1) (Figure 1E). In contrast, the shCD146 2 group displayed low levels of the differentiation marker pan-cytokeratin (CKAE). Msi-1 functions as a transcriptional inhibitor of cell differentiation in colon crypts, while CKAE is a biomarker of epithelial differentiation. Thus, the above result indicates that the differentiation grade in xenografts with low CD146 expression is rather low (Figure 1E).

To further investigate the correlation of CD146 expression with the clinicopathological characteristics of CRC, we performed immunohistochemical staining in a CRC tissue array (90 cases). As shown in Table 1, Pearman correlation analysis showed that CD146 expression in neoplastic cells was significantly associated with the histological grade of CRC (*r* = 0.248, *p* = 0.019). A negative correlation was also observed between CD146 and Duke's stage (*r* = 0.235, *p* =0.026). Together, these findings suggest that, at least in a mouse model, CD146 expression on neoplastic cells suppresses tumorigenesis of CRC, and might be correlated with the degree of differentiation of neoplasm.

**Table 1:** Correlation of CD146 expression with clinical characteristics of CRC

Variables	n	CD146^+^ (n, %)	CD146^−^ (n, %)	*r*	*p*
***Age (years)***					
< 50	4	1 (25.00%)	3 (75.00%)	−0.048	0.656
≥50	86	31(36.05%)	55 (63.95%)		
***Gender***					
Man	47	19 (40.43%)	28(59.57%)	0.128	0.23
Female	43	13 (30.23%)	30 (69.77%)		
***Histological grade***					
G1	9	4(44.44%)	5(55.56%)	0.248	0.019*
G2	71	26(36.62%)	45(63.38%)		
G3	20	2(10%)	18(90%)		
***Duke's stage***					
A	7	4(57.14%)	3(42.86%)	0.235	0.026*
B	49	22(44.90)	27(55.10%)		
C	34	6(17.65%)	28(82.35%)		

***r***: denotes Pearson correlation coefficient

### Knockdown of CD146 in CRC cells restores a stem cell phenotype

**Figure 2:**
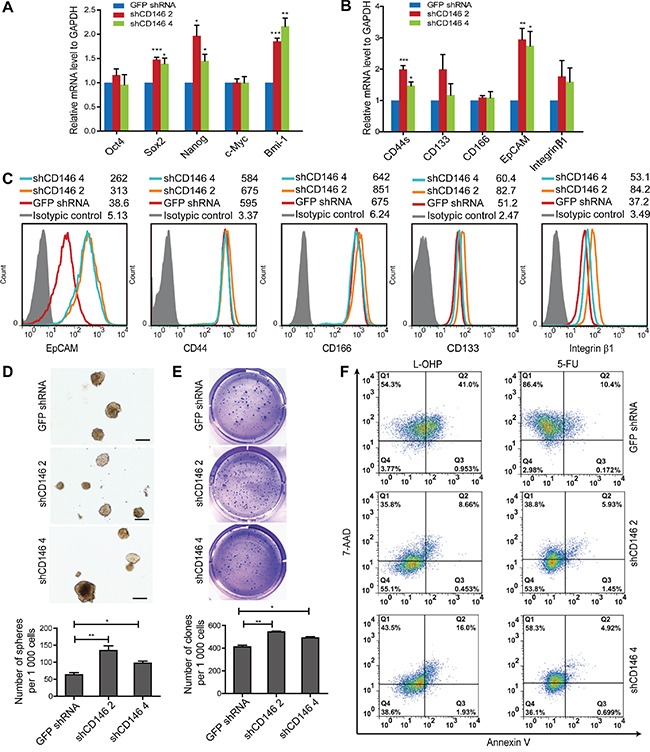
Knockdown of CD146 in CRC cells restores a stem cell phenotype **A.** Knockdown of CD146 upregulates mRNA expression of stemness-related transcriptional factors. **B.** qRT-PCR analysis of putative colorectal CSC surface markers, including CD44 (CD44s, standard form of CD44), CD133, CD166, EpCAM and Integrin β1. **C.** Flow cytometry analysis of CSC markers. Histograms of one representative experiment are shown. **D.** Knockdown of CD146 promotes sphere formation. Representative images of spheres are shown. Scale represents 200 μm. **E.** CD146 knockdown increases clone formation efficiency. Clones in soft agar were counted following crystal violet staining in triplicates. *P<0.05, **P<0.01, ***P<0.001. Error bars, mean ± s.d. **F.** Knockdown of CD146 in CRC cells confers resistance to L-OHP and 5-FU induced apoptosis. Experiments were performed in triplicates, with one representative result shown.

We have shown that CRC cells with CD146 reduction display higher tumor-initiating potential, which is a unique character of CSCs. To investigate whether CD146 inhibits cancer cell stemness *in vitro*. We examined the expression of genes associated with stem cell self-renewal in shRNA transfected cells. qRT-PCR analysis showed that a number of stemness-associated transcription factors, including Sox-2, Nanog and Bmi-1, were upregulated at the mRNA level (Figure 2A, Supplementary Figure S5A). In addition, the mRNA levels of putative colorectal CSC markers, such as CD44 and EpCAM, were also higher in the knockdown groups (Figure 2B). Although the protein expression discrepancies for CD44, CD166, CD133 and Integrin β1 were minor, FACS analysis confirmed the up-regulation of EpCAM at the protein level in knockdown groups (Figure 2C, Supplementary Figure S5B and S5C). Thus, the observed upregulation of stemness-associated gene expression indicates that stem cell phenotypes were restored in CRC cells of CD146 knockdown group.

To further explore the effects of CD146 reduction on stem cell properties, we performed a sphere formation assay, which is widely used as a method to evaluate self-renewal capacity of CSC *in vitro*. In P6C and SW480 fraction cells, knockdown of CD146 significantly increased the efficiency of sphere formation in serum-free medium (SFM) under suspension culture conditions (Figure 2D, Supplementary Figure S5D). This is the hallmark of increased self-renewal ability in CD146 knockdown cells. Anchorage-independent clone formation is a prominent feature of stem cells and is hence used as an indicator for self-renewal and neoplasm formation. We therefore assessed the colon-forming efficiency of CRC cells in a soft agar culture system. Our results showed that upon CD146 knockdown, the clone-forming capacity was enhanced in both P6C cells and SW480 fraction (Figure 2E, Supplementary Figure S5E). Taken together, these findings demonstrate that knockdown of CD146 expression in CRC cells promotes stem cell activity *in vitro*.

Stem cells are resistant to chemotherapy, a fact that is thought to contribute to tumor metastasis and relapse. We thus evaluated the drug susceptibility of these CRC cells following CD146 knockdown. After treatment with either 50μg/ml Oxaliplatin (L-OHP) or 50μg/ml 5-Fluorouracil (5-FU), apoptosis of P6C cells was detected by FACS after Annexin V/7-AAD staining. Compared with the control group, cell viability of CD146 knockdown cells was increased both in the L-OHP (3.77% versus 55.1% and 38.6%) and 5-FU (2.98% versus 53.8% and 36.1%) treatment group (Figure 2F), indicating that knockdown of CD146 results in resistance to apoptosis. Together, our data suggest that CD146 reduction in CRC cells induces stem cell properties, which confer chemoresistance in cancer cells *in vitro*.

### CD146 expression is inversely correlated with Wnt/β-catenin activity in CRC

**Figure 3:**
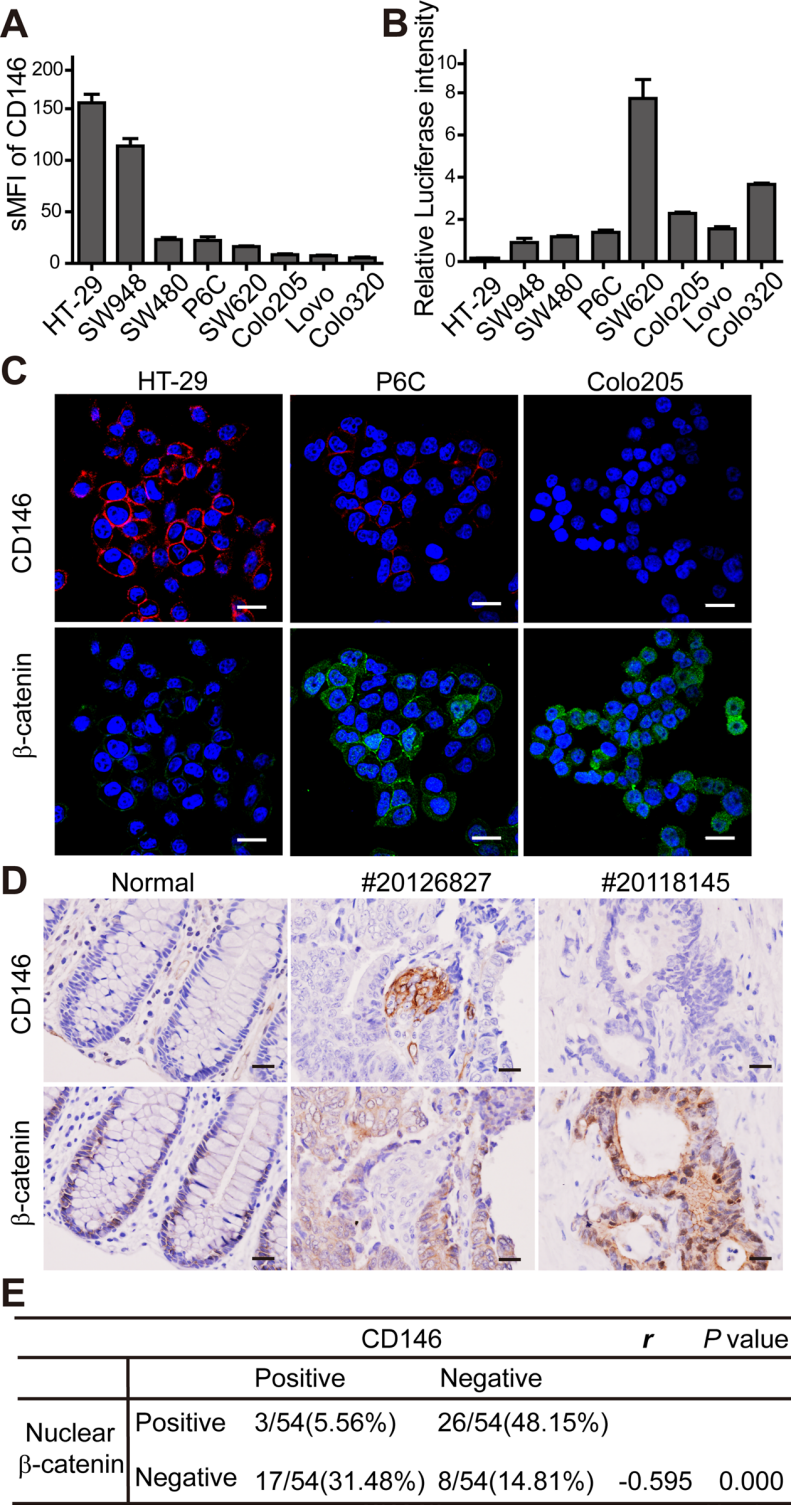
CD146 is inversely correlated with Wnt/β-catenin activity in CRC **A.** FACS analysis of CD146 expression in CRC cell lines. sMFI (specific mean fluorescence intensity) represents the ratio of the mean fluorescence intensity for anti-CD146 antibody over the isotypic control. Error bars, mean ± s.d. **B.** β-catenin/TCF transcription activity of CRC cells, as determined by TOPflash luciferase assay. Relative luciferase intensity denotes the firefly luciferase activity after normalization to Renilla luciferase activity. **C.** Immunostaining of CD146 and β-catenin in CRC cell lines. Cells were counterstained with DAPI (violet). Scale bar represents 20 μm. **D.** Representative immunohistochemistry staining of CD146 and β-catenin in normal human colon tissue and CRC specimens. Scale bar represents 50 μm. **E.** Statistical correlations between CD146 expression and nuclear β-catenin in CRC. Pearson χ2 test was used to determine correlation coefficient (*r*).

Canonical Wnt/β-catenin activity is considered to functionally define the stem cell state in CRC [23]. In our previous study, the inhibitory effect of CD146 on canonical Wnt signaling was observed during zebrafish embryonic development [24]. As presented in Figure 1E, CRC cells with low CD146 expression displayed stronger nuclear staining of β-catenin in xenografts, suggesting that CD146 reduction-induced stemness restoration might be associated with Wnt activity. To investigate the correlation between CD146 expression and Wnt/β-catenin activity in tumor pathogenesis, we tested CD146 expression levels and canonical Wnt activity in a variety of human CRC cell lines, including HT-29, SW948, SW480, P6C, SW620, Colo205, Lovo and Colo320 using FACS analysis. As shown in Figure 3A, HT-29 and SW948 cell lines expressed significantly higher levels of CD146 than SW480 and SW620, whereas CD146 surface expression was barely detectable in Colo205, Lovo and Colo320 cells. In contrast, HT-29 and SW948 cells showed significantly lower Wnt/β-catenin/TCF transcriptional activity compared with those cell lines with low expression of CD146, as determined by TOPflash luciferase reporter assay (Figure 3B). Furthermore, the subcellular localization of β-catenin in CRC cells was examined by immunofluorescence staining. As shown in Figure 3C, β-catenin was present in both the cytoplasm and nucleus of P6C and Colo205 cells with low expression of CD146, but was absent in the nucleus of CD146-overexpressed HT-29 cells. The stabilization and nuclear translocation of β-catenin indicates the activation of canonical Wnt pathway. These observations suggest that in CRC cell lines, low Wnt/β-catenin activity is associated with high expression levels of CD146.

Next, we investigated whether the discrepancy in CD146 expression levels and Wnt activity is reflected in the tumorigenicity of CRC cell lines. We compared the tumorigenecity of two high CD146-expressing cell lines, HT-29 and SW948, with that of the low CD146-expressing cell lines, SW480, SW620 and Colo205 in NOD/SCID mice. Our data showed that Colo205 and SW620 cells were more prone to initiating xenograft tumors (in respect to incidence and latency), especially compared with SW480 and SW948 (Supplementary Figure S6A). SW620 cells initiated tumors in shorter time than HT-29. Colo205 showed advantage than HT-29 in tumor incidence. In addition, we unexpectedly found that HT-29 exhibited higher tumor-initiating capacity than SW948 cell line. Similar trends were observed in the tumor growth curves of these cell lines (Supplementary Figure S6B). Above observations suggest that established CRC cells lines with low CD146 expression levels and high Wnt/β-catenin activity are more tumorigenic *in vivo.* These results imply the negative effect of CD146 on tumorigenesis of CRC cells, which is consistent with our findings in CD146 knockdown experiments (Figure 1). Compared with the artificial gene interference in CRC cells, the distinct cell lines with different mutations and phenotypes better represent the polyclone and heterogeneous hierarchy of tumor entity in patient. Thus, our findings in established CRC cell lines might reflect ever more factually the inhibitory effects of CD146 on β-catenin activity and tumorigenesis in human beings.

To investigate the clinical correlation between β-catenin activity and CD146 expression, we performed immunohistochemistry staining in 54 human CRC specimens. In normal colon tissues, CD146 expression was not detectable in glandular epithelium in normal colon crypts, while the staining of nuclear β-catenin was limited to a few epithelial cells at the bottom of the crypt (Figure 3D). In colorectal carcinoma tissues, CD146 immunoreactivity in neoplastic cells was shown to be variable within a tumor and among different tumors. However, no colocalization of nuclear β-catenin and CD146 was detected in particular neoplasm. As shown in Figure 3D for tumor #20126827, membrane staining of CD146 was detected in a small number of neoplastic cells, while β-catenin was exclusively expressed in the membrane and cytoplasm of neoplastic cells lacking CD146 expression. In contrast, cells exhibiting intense staining of nuclear β-catenin were negative for CD146 expression (as shown for tumor #20118145). Among all of the 54 carcinoma samples, nuclear β-catenin was detected in 48% of CD146-negative samples, while it was only found in 6% of CD146-positive samples (Figure 3E). In comparison, CD146 expression was detected in a higher proportion of cases without nuclear β-catenin staining (~31 %) relative to those with nuclear β-catenin staining (~6%). Correlation analysis using Pearson χ^2^ test showed that the presence of nuclear β-catenin was negatively correlated with CD146 expression in neoplastic cells (r = −0.059). Taken together, these results show a strong negative correlation between CD146 expression and β-catenin activity in both CRC cell lines and primary tumor tissues.

### Knockdown of CD146 activates canonical Wnt signaling in CRC cells

**Figure 4:**
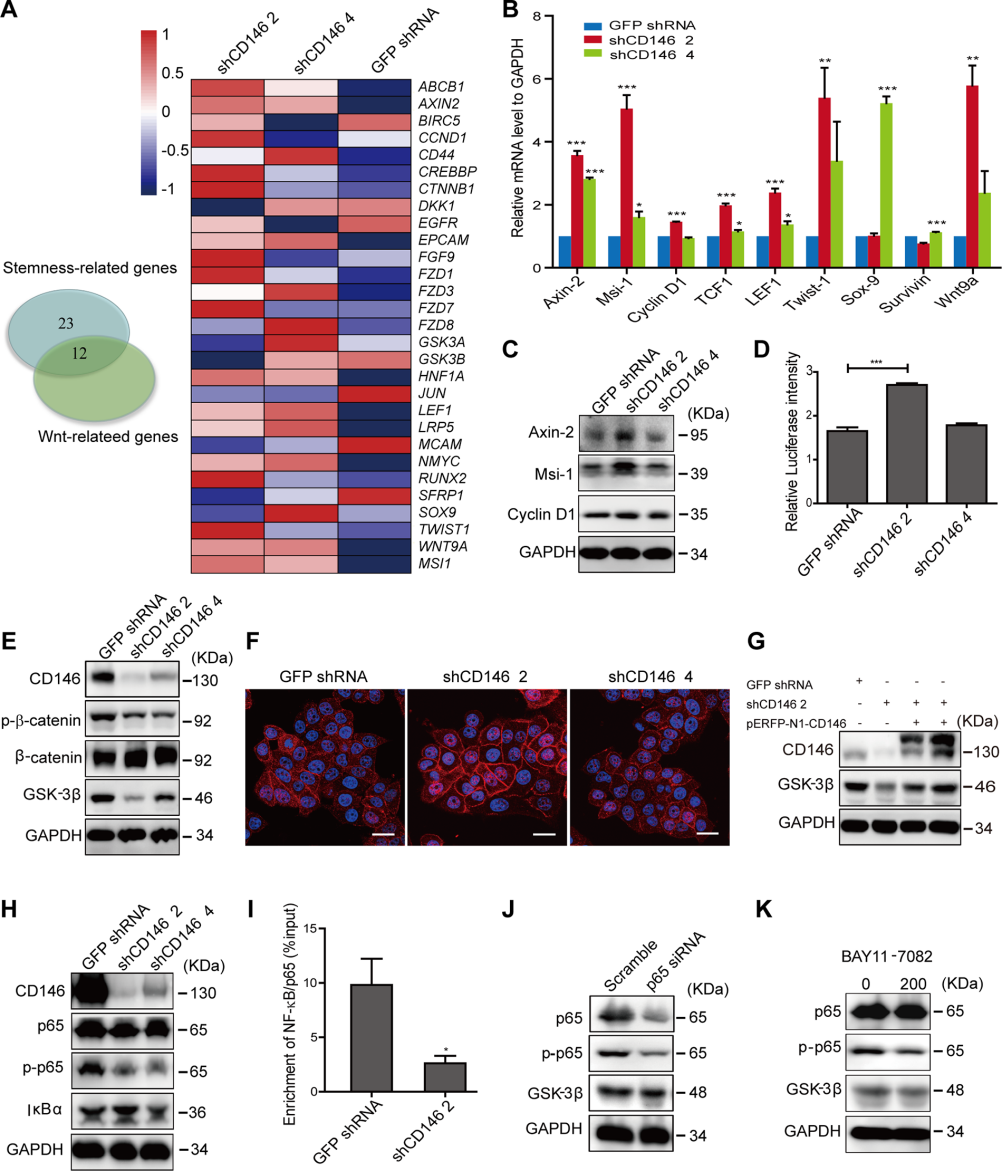
Knockdown of CD146 activates canonical Wnt signaling in CRC cells **A.** Differential gene expression upon CD146 knockdown in P6C cells. Left: Venn diagram showing the number of differentially expressed genes associated with stemness and Wnt signaling. Genes were identified based on the GO term analysis of microarray data. Right: Differential expression of Wnt-associated genes, as represented in the heatmap. **B.** Upregulation of Wnt target genes in CD146 knockdown cells was validated by qRT-PCR analysis. **C.** Western blotting analysis of Wnt target genes in CD146 knockdown cells. GAPDH was used as a loading control. **D.** Knockdown of CD146 promotes β-catenin/TCF transcription activity, as determined by Dual-Luciferase reporter assay. **E.** Knockdown of CD146 influences Wnt-downstream signaling elements. **F.** Immunostaining for β-catenin in P6C cells. Scale bar represents 20 μm. **G.** Rescued CD146 expression up-regulates the protein level of GSK-3β. CD146 knockdown cells were transfected with *pERFP-N1-CD146* expression plasmid. **H.** Knockdown of CD146 represses the NF-κB/p65 signaling activity. **I.** Binding of NF-κB/p65 to the predicted site in GSK-3β gene promoter was validated by ChIP assays. NF-κB/p65 enrichment was normalized to input control. **J-K.** GSK-3β expression was inhibited by both transfection of p65-specific siRNA and treatment with NF-κB inhibitor BAY11-7082 (200 ng/ml). *P<0.05, **P<0.01, ***P<0.001. Error bars, mean ± s.d.

To elucidate the precise mechanisms underlying the inhibitory effects of CD146 on cancer stemness, we performed differential gene expression analysis. Whole-genome gene expression of shCD146-transfected monoclones of P6C was profiled using Affymetrix Human U133 Plus 2.0 Microarrays, following by Gene Ontology (GO) term annotation analysis. Pathway analysis showed that numerous genes involved in stemness-associated pathways, such as Wnt, Notch and Hedgehog pathways, were influenced by CD146 knockdown (Supplementary Table S1). We have observed a negative correlation between Wnt/β-catenin activity and CD146 in CRC cells. In addition, canonical Wnt signaling facilitates colorectal carcinogenesis and stem cell self-renewal, as reported in previous work. Thus, we speculated that a reduction of CD146 expression restores stem cell phenotype in CRC cells through reactivating Wnt/β-catenin signaling. To test this hypothesis, we performed GO term enrichment analysis, which showed that 35 differentially expressed genes are involved in stemness regulation. Among those 35, 12 genes were also associated with Wnt signal transduction (Figure 4A, Supplementary Table S2). As shown in the heat map in Figure 4A, a large number of Wnt-associated genes were differentially expressed in CD146 knockdown cells. The increase in expression of Wnt target genes, such as *Axin-2*, *Msi-1*, *Cyclin D1* (also known as *CCND1*), *TCF1* (also known as *HNF1A*) and *LEF1*, were validated by qRT-PCR in shCD146-transfected P6C cells (Figure 4B). Similarly, *Axin-2* and *Cyclin D1* were found to be significantly upregulated when CD146 was knocked down in the SW480 fraction (Supplementary Figure S7A). Western blot analysis further confirmed that the protein expression of *Axin-2* and *Msi-1* was upregulated in shCD146 2 group (Figure 4C, Supplementary Figure S12). Furthermore, the TOPflash luciferase reporter assay showed that β-catenin/TCF transcriptional activity was increased in CD146 knockdown cells (Figure 4D).

We next elucidated mechanism by which CD146 inactivates the canonical Wnt signaling pathway. We found that the phosphorylation of β-catenin at Ser33, Ser37 or Thr41 was attenuated, with a simultaneous increase observed in the protein level of total β-catenin upon CD146 knockdown (Figure 4E, Supplementary Figure S7B, Supplementary Figure S12). We also observed a strong increase in cytosolic accumulation and nuclear translocation of β-catenin in shCD146 2 group (Figure 4F). Given that phosphorylation of β-catenin results in its degradation in the cytoplasm, these observations strongly indicate that CD146 suppresses Wnt activity by decreasing intracellular level of free β-catenin. Since β-catenin is ultimately phosphorylated by GSK-3β, we next examined the cellular activity of GSK-3β. As shown in Figure 4E, the expression of GSK-3β was dramatically attenuated upon CD146 knockdown in P6C and SW480 fraction (Supplementary Figure S7B). Moreover, protein level of GSK-3β were restored when the expression of CD146 was rescued in knockdown cells (Figure 4G, Supplementary Figure S7C, Supplementary Figure S12). This reciprocal relation indicates that GSK-3β expression is increased by the presence of CD146. We therefore focused on elucidating the molecular basis by which CD146 promotes the expression of GSK-3β. Earlier, it was reported that anti-CD146 monoclonal antibody blocks the conditioned medium-induced NF-κB/p65 activation in endothelial cells [45]. Here, we found that CD146 itself triggers the NF-κB/p65 signaling cascade in CRC cells. Knockdown of CD146 resulted in the decreased phosphorylation of NF-κB/p65, indicating the inactivation of NF-κB signaling (Figure 4H, Supplementary Figure S7D, Supplementary Figure S12). Using three online programs (AliBaba2.1, JASPAR and ECR), we identified a potential NF-κB/p65 binding site at 76~85bp (GGGGAAGTCC) upstream of the transcription start site of *GSK-3β*. This predicted binding site was verified experimentally by chromatin immunoprecipitation (ChIP) assays, which showed a decrease in enrichment for NF-κB/p65 at this site upon CD146 knockdown (Figure 4I, Supplementary Figure S7E). Moreover, blockage of NF-κB/p65 by either specific siRNA (Figure 4J, Supplementary Figure S7F, Supplementary Figure S12) or NF-κB inhibitor BAY11-7082 (Figure 4K, Supplementary Figure S7G, Supplementary Figure S12) attenuated the expression of GSK-3β, indicating that NF-κB/p65 is indeed a transcription activator of GSK-3β. Quantitative analysis of protein expression using western blotting are shown in Supplementary Figure S12. These results suggest that CD146 reduction activates β-catenin through down-regulating NF-κB/p65-initiated GSK-3β expression.

**Figure 5:**
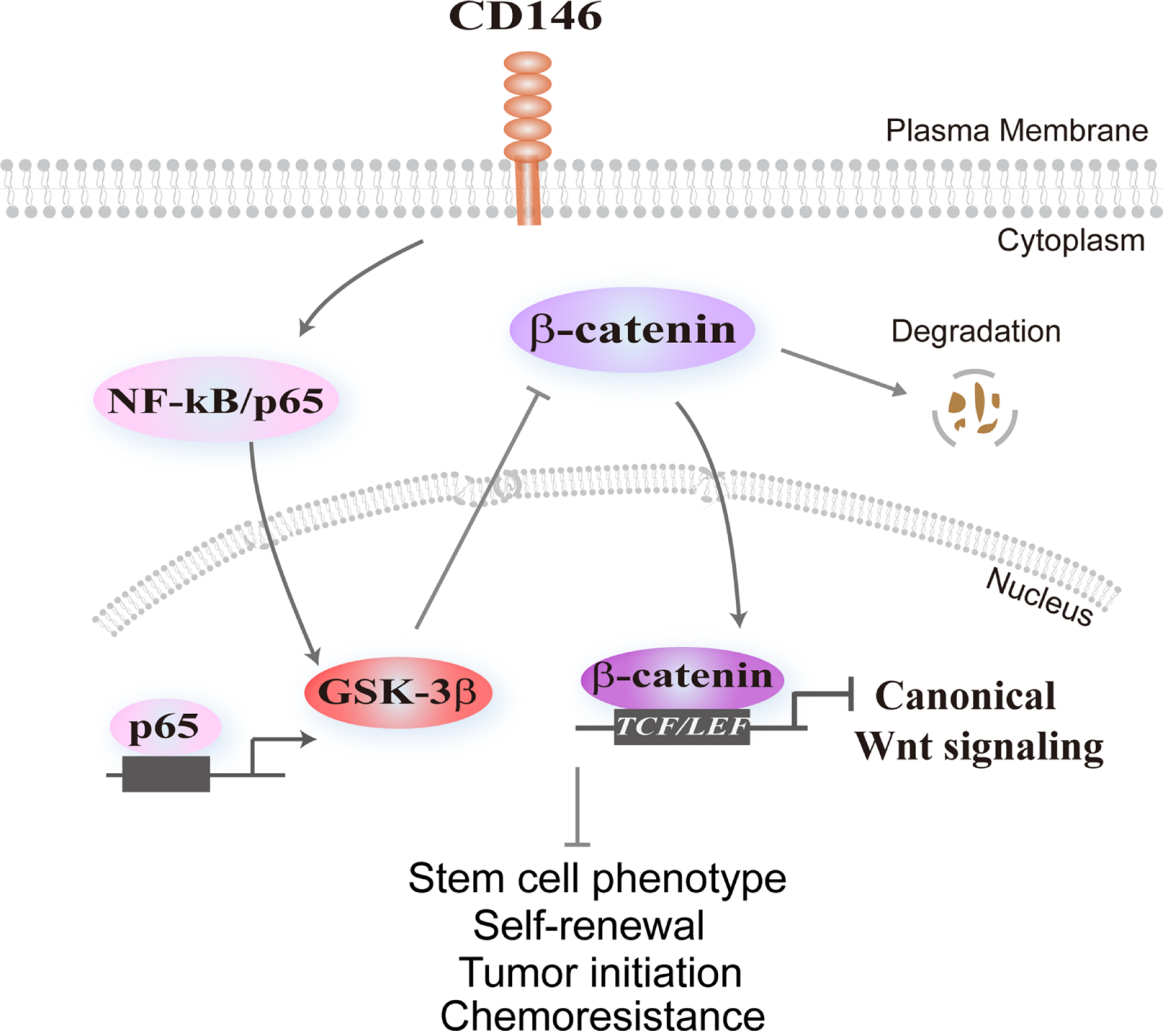
Schematic model illustrating the effect of CD146 on canonical Wnt signaling and cancer stemness CD146 triggers nuclear translocation of NF-κB/p65, resulting in transcriptional activation of GSK-3β. Elevated GSK-3β levels promote β-catenin phosphorylation and its subsequent destabilization, attenuating nuclear translocation of β-catenin and subsequent TCF/LEF-initiated transcription of Wnt target genes. Conversely, reduction of CD146 expression in CRC cells restores the activity of Wnt/β-catenin, leading to the reprogramming of differentiated CRC cells towards to the stem-like state characterized by tumor-initiating, self-renewal capacity and chemoresistance.

In conclusion, we elucidate the molecular basis underlying CD146 reduction-induced restoration of stem cell characteristics in CRC cells (as illustrated in Figure 5). CD146 facilitates the degradation of β-catenin by promoting NF-κB/p65-initiated GSK-3β expression, and thus subsequently leads to inactivation of the canonical Wnt/β-catenin signaling pathway. The reduced Wnt/β-catenin activity causes the gradually depletion of cancer cell stemness in CRC [23]. Conversely, the reduction of CD146 in CRC cells facilitates the transcriptional activation of β-catenin/TCF/LEF complex and thus endows CRC cells with stem cell phenotype, as well as enhanced self-renewal, tumor initiating capacity and chemoresistance.

## DISCUSSION

It was established earlier that CD146 participates in tumor angiogenesis and metastasis [29, 46]. Here, we report for the first time that CD146 suppresses cancer stemness and tumorigenesis of CRC through inactivating Wnt/β-catenin signaling pathway.

Our *in vitro* and *in vivo* studies supported the hypothesis that CD146 acts a negative regulator of cancer cell stemness in CRC. We found that CD146 reduction results in the upregulation of EpCAM, as well as pluripotency-associated transcription factors. EpCAM was initially identified in CRC cells and has been supposed to provide nuclear signaling sustaining CSC [47]. It has been reported previously that EpCAM^high^CD44^+^ is a more robust marker profile for colorectal CSC characterization than earlier identified CD44^+^CD133^+^ combination [6, 48]. CD146 knockdown cells with EpCAM^high^CD44^+^ phenotype showed significant advantages in tumor formation and chemoresistance over the control group. It is therefore conceivable that CD146 reduction induces the reprogramming of differentiated CRC cells towards more tumorigenic stem-like cells. These results might provide new evidence for the plasticity of cancer cells [7, 8]. Besides the gene interference, we also found that CD146 is down-regulated in spheroid cultures under SFM condition compared with adherent P6C cells maintained in 10% fetal bovine serum (Supplementary Figure S9). Moreover, CD146 expression decreases in P6C cells under hypoxic conditions (Supplementary Figure S10). It has been reported earlier that serum-free and hypoxic condition both prevent differentiation and maintain the stem cell state [49, 50]. Therefore, above observations provide additional evidence for the negative correlation between CD146 expression level and stemness of CRC cells. It is noteworthy that the enforced expression of CD146 in breast cancer cells triggers epithelial-mesenchymal transition (EMT), which induces stem cell properties in breast cancer cells [32]. In our study, CD146 suppresses stem cell properties and epithelial phenotypes in CRC cells (as characterized by increased EpCAM and E-cadherin expression, Supplementary Figure S11). These seemingly conflicting results imply that the precise effects of CD146 on cancer stemness might depend on the exact tumor type.

On the basis of our results, we propose a mechanistic model for the inhibitory effect of CD146 on cancer stemness in CRC. Under physiological conditions, the Wnt pathway typically remains in the off state due to the absence of corresponding ligands or the involvement of Wnt antagonists. In our study, CD146 itself acts as a suppressor of canonical Wnt signaling. Previous study reported that GSK-3β promotes NF-κB activity in colon cancer [51]. Here, we reveal that CD146-initiated NF-κB activation promotes GSK-3β expression, which might drive a positive feedback loop and ultimately facilitates β-catenin degradation. Therefore, reduced CD146 might relieve the inhibitory effect of GSK-3β on β-catenin activity, and thus induce dedifferentiation of CRC cells. However, although we did take caution about the choice of cell lines for testing, we could not exaggerate the role of CD146 in all types of colorectal cancer cells, especially for the cells carrying numerous mutations. The constitutive activation of canonical Wnt pathway caused by β-catenin mutations might be CD146-independent. Such expectations are somewhat reflected by the CD146 and nuclear β-catenin double positive specimens in tissue array (Figure 3E). These findings might benefit the personalized drug development and cancer therapy.

Our findings, together with previous reports reveal the dual effects of CD146 on CRC progression. It was reported earlier that aberrant expression of CD146 on neoplastic cells promotes CRC metastasis [52]. Here, we found that CD146 suppresses tumorigenesis of CRC in NOD/SCID mice. These conflicting roles might be due to the different regulatory modes of CD146 on the two branches of Wnt pathway. Apart from its inhibition of canonical Wnt activity, CD146 simultaneously activates the non-canonical Wnt/JNK pathway in CRC cells (Supplementary Figure S8), which is consistent with our previous findings [24]. Hyperactivation of canonical Wnt signaling is an early critical event during the carcinogenesis of CRC [19]. On the contrary, the non-canonical cascade is found to inhibit malignant transformation in the early stage but promote invasion in the late stage of CRC [53, 54]. It is therefore conceivable that the canonical and non-canonical cascades might be dominant during the different stages of CRC. Thus, the distinct effects of CD146 on two branches might provide a rational explanation for its dual roles in tumor initiation and metastasis of CRC. Moreover, CD146 expression on lymphocytes and endothelial cells also contributes to CRC progression [55]. Therefore, the effects of CD146 on the clinical outcome of CRC might be comprehensive and should be evaluated with caution.

Taken together, our study revealed that CD146 acts as a negative regulator for canonical Wnt/β-catenin signaling and cancer stemness in CRC. The results presented here provide a better understanding for the determinant and molecular basis sustaining stem cell plasticity, and also provide new insights into the multifaceted functions of CD146 in CRC progression. These findings should benefit the development of novel approaches to diagnose or treat CRC in the future.

## MATERIALS AND METHODS

### Cell culture

CRC cell lines (HT-29, SW948, SW480, SW620, Colo205, Lovo, and Colo320) were purchased from ATCC and cultured in DMEM medium supplemented with 10% FBS and 100μg/ml penicillin/streptomycin at 37°C and 5% CO_2_. Primary cell line P6C and CD44^+^CD133^+^ SW480 Fraction cells were maintained in DMEM containing 2% FBS and sub-cultured within 20 passages *in vitro.*

### shRNA and siRNA transfection

GFP-expressing lentiviral particles with CD146-targeting shRNA constructs in GV248 lentiviral vector were packaged in 293T cells (Genechem Technologies, Shanghai). Non-effective GFP shRNA was used as a negative control. The sequences and respective cDNA locations of shCD146 1, 2, 3 and 4 are shown in Supplementary Table S3. shRNA transfection and establishment of stably transfected monoclones (shCD146 2 and shCD146 4) were performed as directed by the manufacturer. Transient transfection with NF-κB/p65 siRNA (Cell Signaling Technology) or *pERFP-N1-CD146* plasmid were performed using Fugene-6 transfection reagent (Roche) following the manufacturer's instructions.

### Serial xenotransplantation assay

Single CRC cells in PBS mixed with Matrigel (1:1, BD Biosciences) were injected subcutaneously into NOD/SCID mice (female, 6-week-old, 5 mice per group). Tumor formation and growth were monitored every three days. Tumor sizes were measured by the formula: (length×with^2^)/2. Mice were sacrificed at three months after transplantation or until the tumor overburden. For the secondary transplantations, first xenografts generated in were dissociation with Collagenase Type IV (GIBCO), GFP-positive cell subpopulations with high, medium and low CD146 expression levels were sorted from GFP shRNA, shCD146 2 and shCD146 4 groups. Isolated cells were injected into the secondary recipients as performed in the first transplantation. All of the animal experiments were approved by the Animal Care and Ethical Committee of the Institution of Biophysics.

### Immunohistochemistry staining

CRC tissue arrays (90 cases) were purchased from Shanghai Outdo Biotech Co., Ltd. (Shanghai, China). CRC specimens were obtained from Capital Medical University Affiliated Beijing Anzhen Hospital (Beijing, China). All samples were obtained with patient consent and approvals from the Ethics Committee of Anzhen Hospital and the Institute of Biophysics. Immunohistochemistry was performed as described [56]. Anti-CD146 (AA4), anti-β-Catenin (BD Biosciences), anti-Msi-1(Cell Signaling Technology) and anti-pan-Cytokeratin (CKAE) antibody (Dako) were used. Positive staining for CD146 or nuclear β-Catenin were defined as the following criteria: the percentage of reacting neoplastic cells was higher than >10% (counting at least 1000 cells).

### Quantitative RT-PCR

Quantitative RT-PCR (qRT-PCR) was performed with SYBR Green (Invitrogen) following the manufacturer's instructions on the Corbett Rotor-gene 6200 Real-Time PCR system. All primers were listed in Supplementary Table S4.

### Flow cytometry analysis and cell sorting

Single cell suspensions were incubated with primary antibodies and isotypic controls, followed by Alexa Fluor dyes-conjugated secondary antibodies, or stained with fluorescence-conjugated primary antibodies: anti-EpCAM-PerCP-CY5.5 (BD Biosciences), anti-CD44-APC (BioLegend), anti-CD133/1-PE (Miltenyi Biotec), anti-CD166-PE (R&D Systems), anti-CD146 monoclonal antibody (AA1, made in our laboratory). Flow cytometry analyses were performed using BD FACS Calibur. Cell sorting was conducted using FACS Aria Cell Sorting System (BD).

### Sphere and clone formation assay

In sphere formation assay, cells were plated in 6-well ultra-low adherent plates (Corning) at a density of 1×10^3^ cells/well. Spheres were maintained in serum-free medium (SFM: DMEM/F12, supplemented with penicillin/streptomycin, non-essential amino acids, sodium pyruvate, B27, heparin, N2 supplement, 20 ng/ml EGF and 10 ng/ml bFGF) for two weeks. Spheres were checked with a fluorescence microscope. Clone formation was carried out as described [56].

### Chemoresistant assay

Cells were seeded into 6-well plates at a density of 1×10^6^ cells/well. After incubation for 24h, cells were treated with Oxaliplatin (L-OHP, 50 μg/ml) or 5-Fluorouracil (5-FU, 50 μg/ml) for 24h. All of the Cells were harvested and labeled with Annexin V/7-AAD as described in the Annexin V apoptosis kit manual (Tianjin Sugene, China). Cell apoptosis was analyzed by flow cytometry.

### Luciferase reporter assay

Colorectal cancer cells were seeded in 48-well plates. Transfection of TCF reporter plasmid (30ng of Super *TOPflash* containing TCF response elements, 1ng of control *pRL-TK*) was performed as described [24]. Firefly luciferase and Renilla luciferase activity were measured by the GloMax-Multi Detection System (Promega) following the manufacturer's instructions.

### Immunofluorescence

Immunofluorescence assay was carried out as previously described [24]. The images were captured by the Olympus FluoView Confocal Microscope.

### Microarray analysis

Gene expression of stably shCD146-transfected P6C monoclones was profiled using Affymetrix Human U133 Plus 2.0 Microarrays (Shanghai Biotechnology). RNA extraction, cDNA preparation, hybridizations and scaning were conducted in accordance with the manufacturer's instructions (Affymetrix).

### Western blottings

Western blottings were performed as described [45]. Antibodies used included anti-phospho-β-Catenin (Ser33/Ser37/Thr41), anti-GSK-3β, anti-Axin-2, anti-phospho-JNK, anti-NF-ĸB/p65, anti-phospho-NF-ĸB/p65 and anti-IĸBα, which were purchased from Cell Signaling Technology, anti-Cyclin D1 (Santa Cruz) and anti-GAPDH antibody (Abcam). Specific bands were visualized with the West Dura Extended Duration Substrate (Pierce) and Chemiluminescence Analysis System (ChemiScope Series, Clinx). The cropped blots were displayed in the figures. The intensities of bands were measured by the ChemiScope Analysis System.

### Chromatin immunoprecipitation (ChIP) assays

ChIP assays were performed as described [57]. Anti-NF-ĸB/p65 (Abcam) and rabbit IgG (California Bioscience) were used. Purified DNA was assayed by the real-time qPCR using the primers specific for potential chromatin binding sites of NF-κB/p65 (Supplementary Table S5).

### Statistical analysis

Results were represented as mean ± s.d. value. Statistical differences were determined with two-tail unpaired student's *t*-test using GraphPad Prism 5 software. Significance of differences in tumor growth curves were assessed by two-way ANOVA analysis. Correlation analysis was conducted with Pearson χ2 test using SPSS software. Statistical significance was defined as *P<0.05, **P<0.01, ***P<0.001.

## SUPPLEMENTARY FIGURES AND TABLES


