# Supplementary material for: Reduced CD146 expression promotes tumorigenesis and cancer stemness in colorectal cancer through activating Wnt/β-catenin signaling
Source: Oncotarget. 2016 Jun 9;7(26):40704–18. doi: 10.18632/oncotarget.9930 (PMC5130037; doi:10.18632/oncotarget.9930)
Supplement: Supplementary file 1 [file oncotarget-07-40704-s001.pdf]

Reduced CD146 expression promotes tumorigenesis and cancer stemness in colorectal cancer through activating Wnt/β-catenin signaling

SUPPLEMENTARY FIGURES AND TABLES

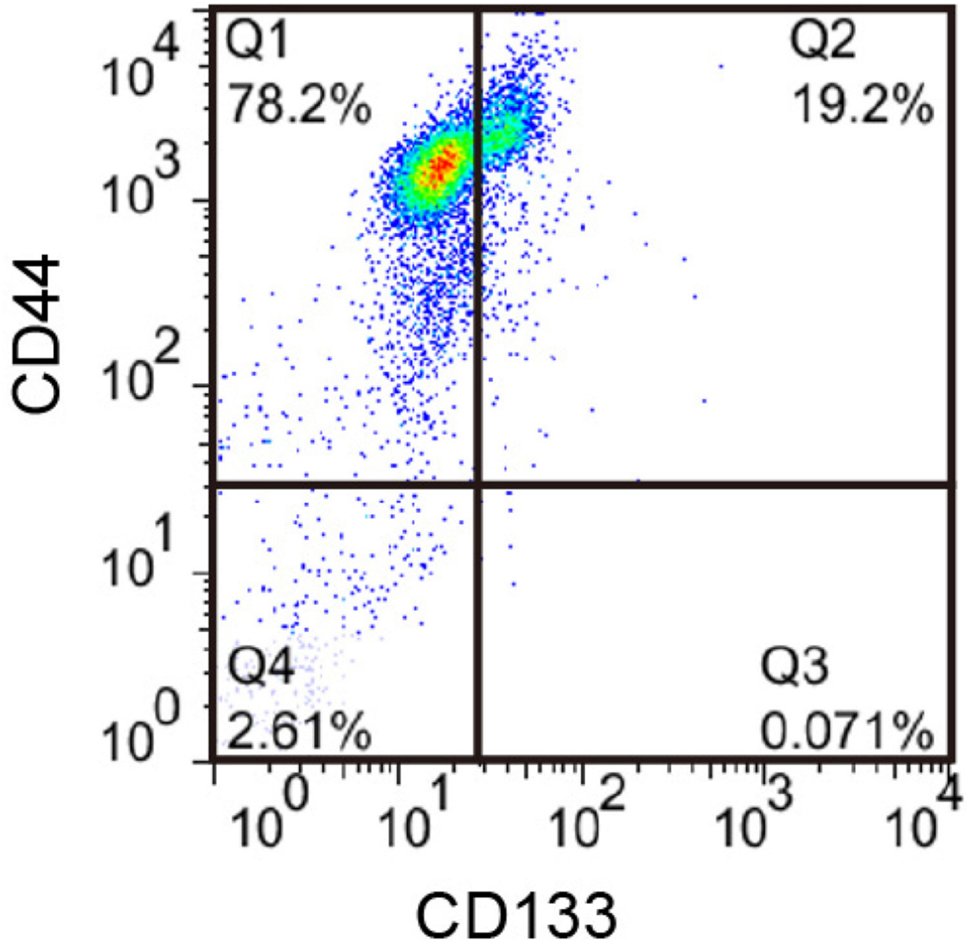

Supplementary Figure S1: Phenotypic identification of primary CRC cell line P6C. FACS analysis of CD44 and CD133 expression on P6C cells.

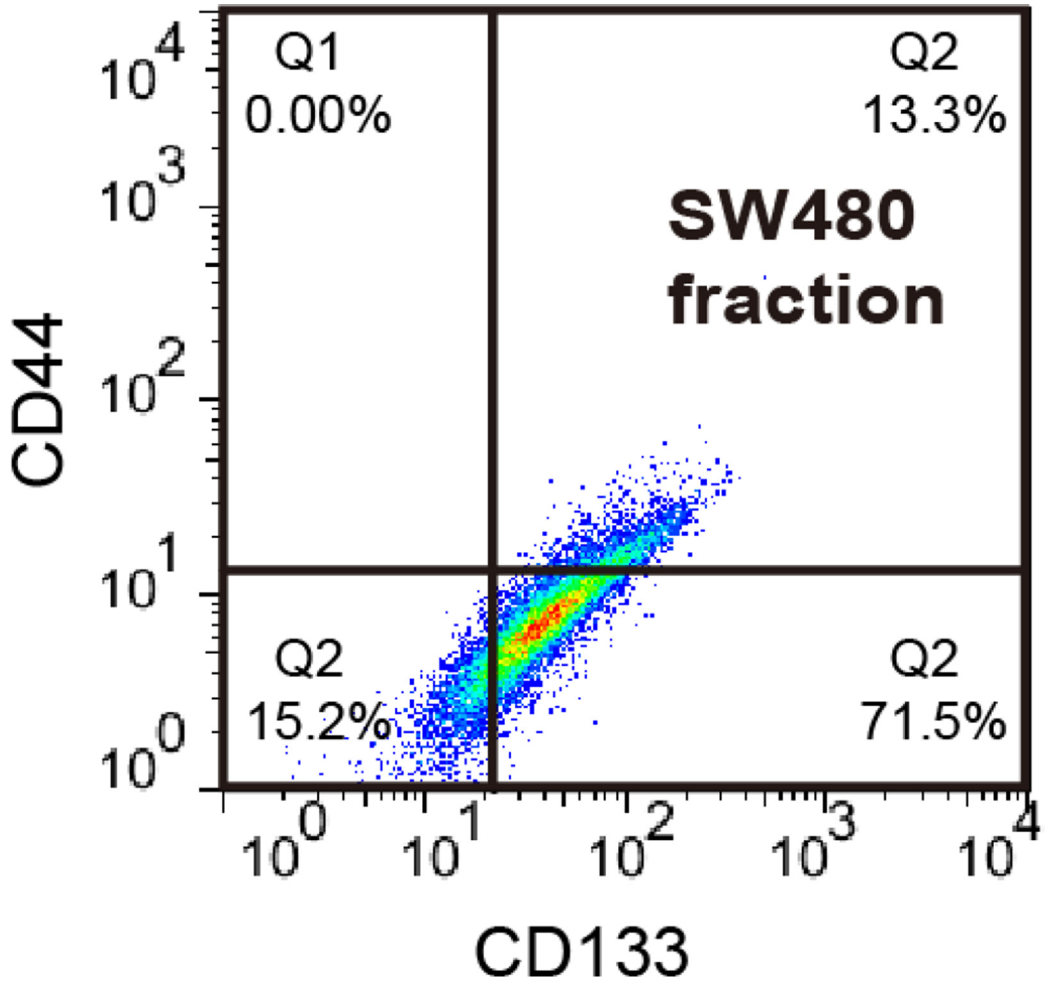

Supplementary Figure S2: FACS sorting of SW480 fraction based on the expression of CD44 and CD133. SW480 fraction denotes the $CD44^{+}CD133^{+}$ subset in established CRC cell line SW480.

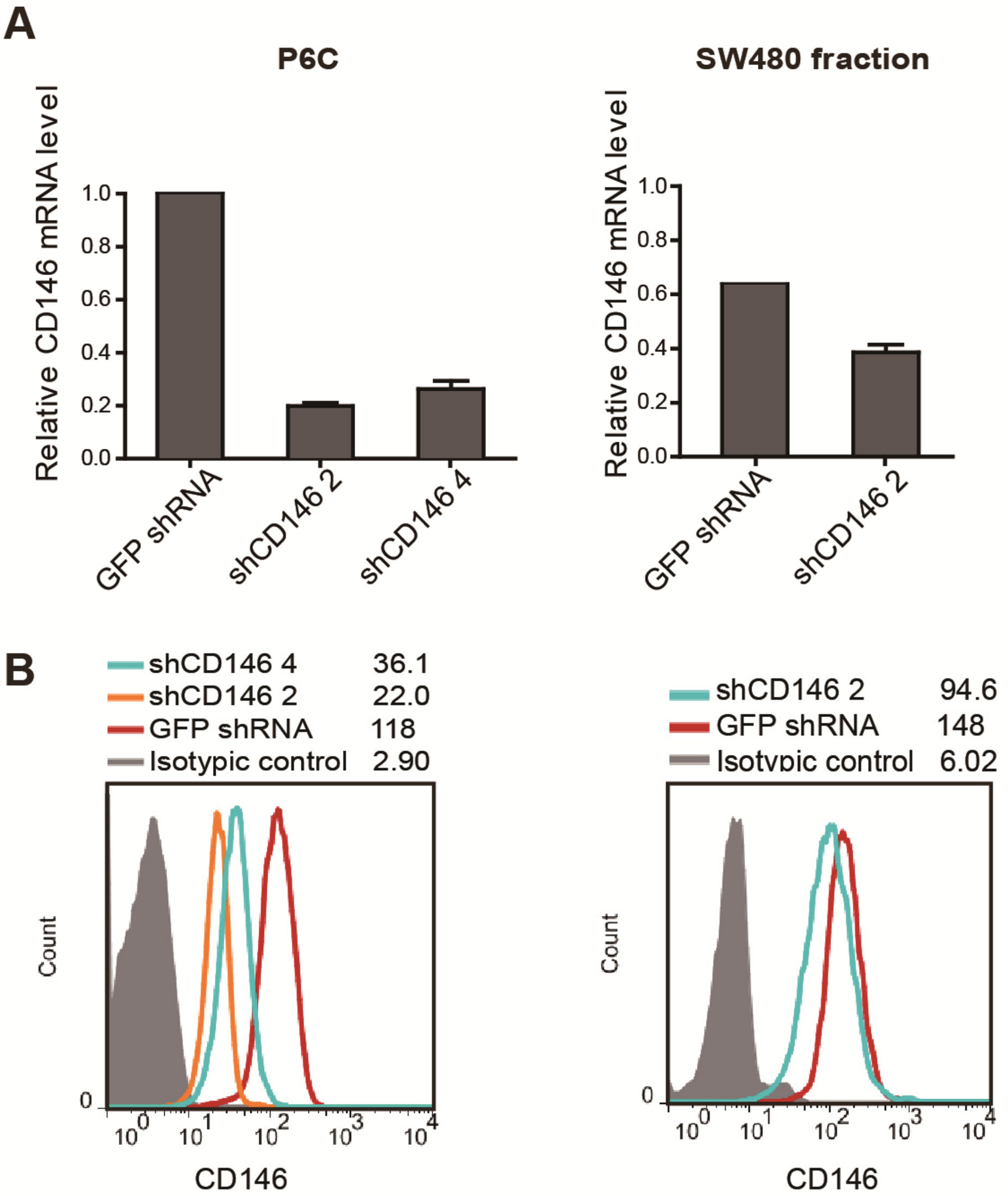

Supplementary Figure S3: Knockdown efficiency of CD146 in P6C and SW480 fraction with shRNAs transfection. **A.** The mRNA expression levels of CD146 in P6C cells and SW480 fraction, as assessed by qRT-PCR analysis. GFP shRNA was used as a negative control. The relative mRNA level of CD146 was normalized to human GAPDH mRNA expression. Error bars, mean ± s.d. **B.** The protein levels of CD146 in P6C cells (left) and SW480 fraction (right) were analyzed by FACS. Histograms of one representative experiment are shown.

A

	Cell subset	Incidence	Latency
1°	GFP shRNA	2/5	32
	shCD146 2	4/5	19
2°	GFP shRNA	1/5	45
	shCD146 2	2/5	23

Notes: 1 000 single cells were injected .

B

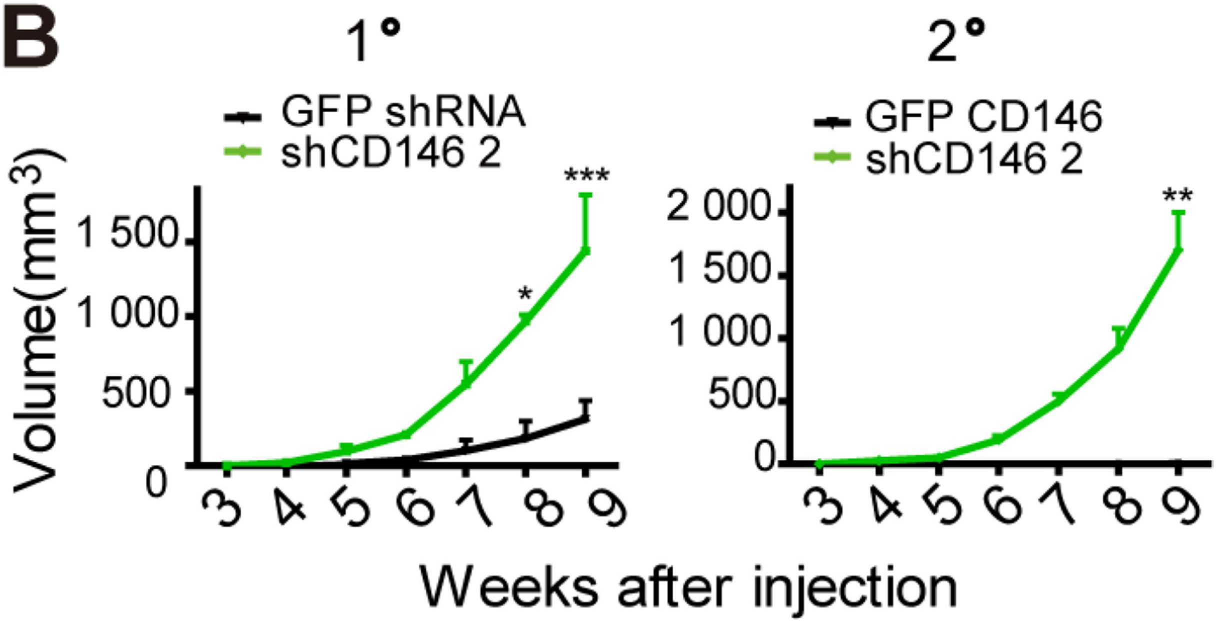

Supplementary Figure S4: Reduced CD146 expression promotes colorectal tumorigenesis *in vivo*. A. Reduced expression of CD146 facilitates tumor formation in serial transplantations. 1000 SW480 fraction cells were used for each injection (n=5). **B.** Tumor volumes and growth curves were monitored in serial recipients. Significance of differences at indicate time point were determined by two-way ANOVA analysis, **P<0.05, **P<0.01, ***P<0.001.* Error bars, mean ± s.d.

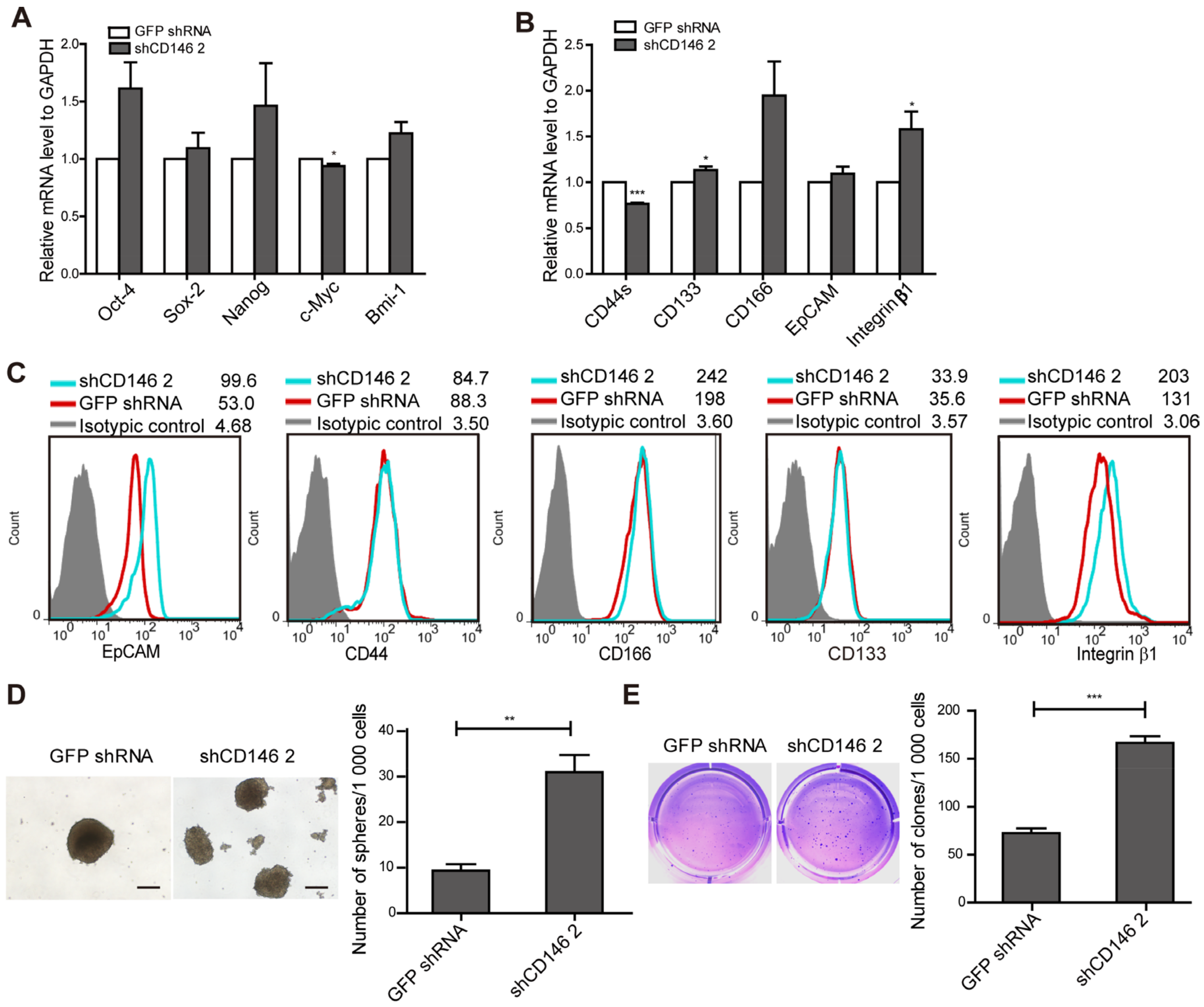

Supplementary Figure S5: Knockdown of CD146 in SW480 fraction restores a stem cell phenotype. A. The mRNA expression levels of stemness-related transcription factors in SW480 fraction, as determined by qRT-PCR. Data were expressed as mean value ± s.d. of three independent experiments. **B.** The mRNA expression levels of colorectal CSC markers in SW480 fraction. **C.** FACS analysis of CSC surface markers. **D.** CD146 knockdown promotes sphere formation. 1000 SW480 fraction cells were cultured in ultra low adherent plates in triplicates under SFM condition. **E.** CD146 knockdown increases clone formation efficiency. **P<0.05, **P<0.01, ***P<0.001*. Error bars, mean ± s.d.

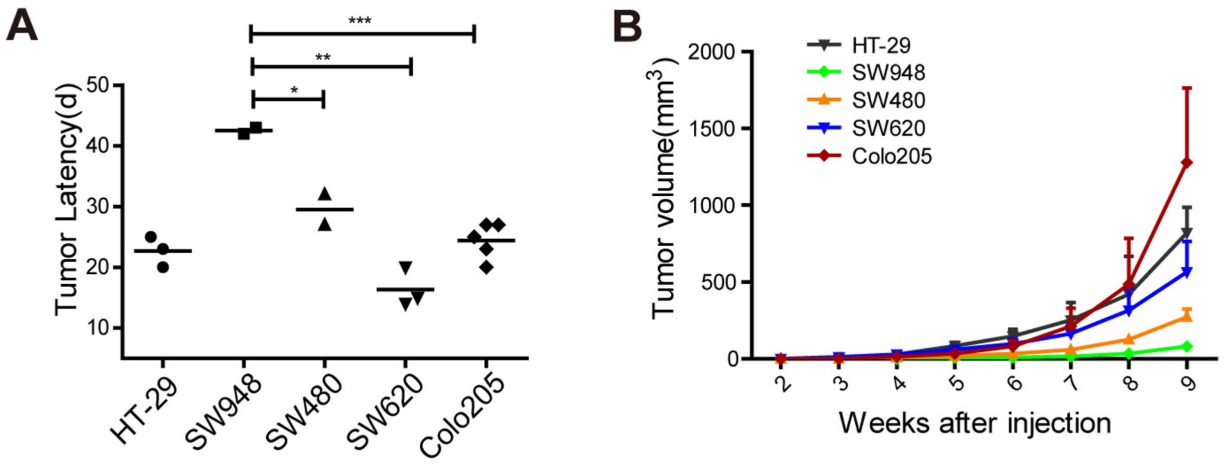

Supplementary Figure S6: Tumorigenicity of five established human CRC cell lines *in vivo*. A. Tumor incidence and latency in xenotransplantation. 1000 cells were used for each subcutaneous injection to NOD/SCID mice (n=5). **B.** Tumor volumes and growth curves were monitored and measured weekly. **P<0.05,*P<0.01, ***P<0.001*. Error bars, mean ± s.d.

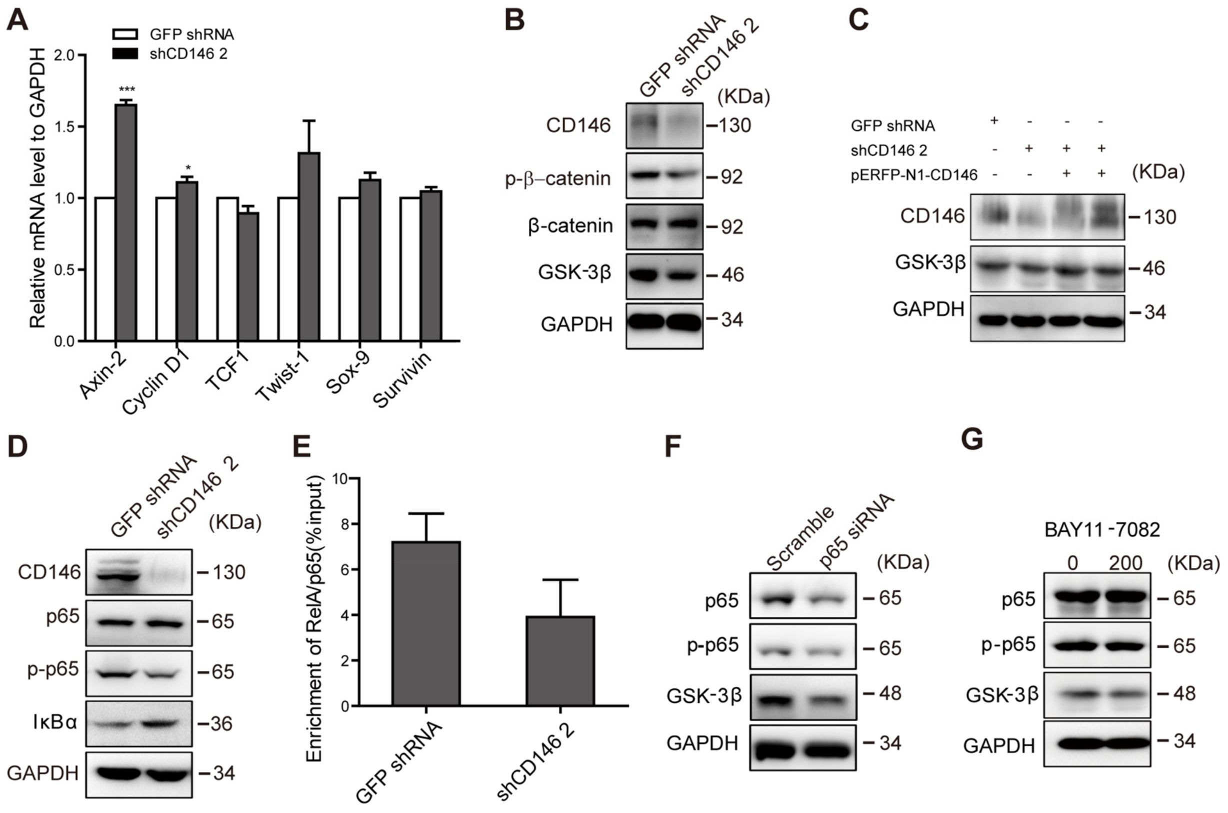

Supplementary Figure S7: Knockdown of CD146 activates canonical Wnt signaling in SW480 fraction cells. A. The mRNA expression levels of Wnt target genes upon CD146 knockdown. Data were expressed as mean value ± s.d. of three independent experiments. **B.** Knockdown of CD146 represses β-catenin phosphorylation and GSK-3β expression. GAPDH were used as a loading control. **C.** Rescued CD146 expression up-regulates the protein level of GSK-3β. **D.** Knockdown of CD146 inhibits NF-κB/p65 signaling pathway. **E.** Binding of NF-κB/p65 to the predicted site in GSK-3β gene promoter was validated by ChIP assays. NF-κB/p65 enrichment was normalized to input control. **F-G**. GSK-3β expression was inhibited by both transfection of p65-specific siRNA and treatment with NF-κB inhibitor BAY11-7082 (200 ng/ml). **P<0.05, ***P<0.001*. Error bars, mean ± s.d.

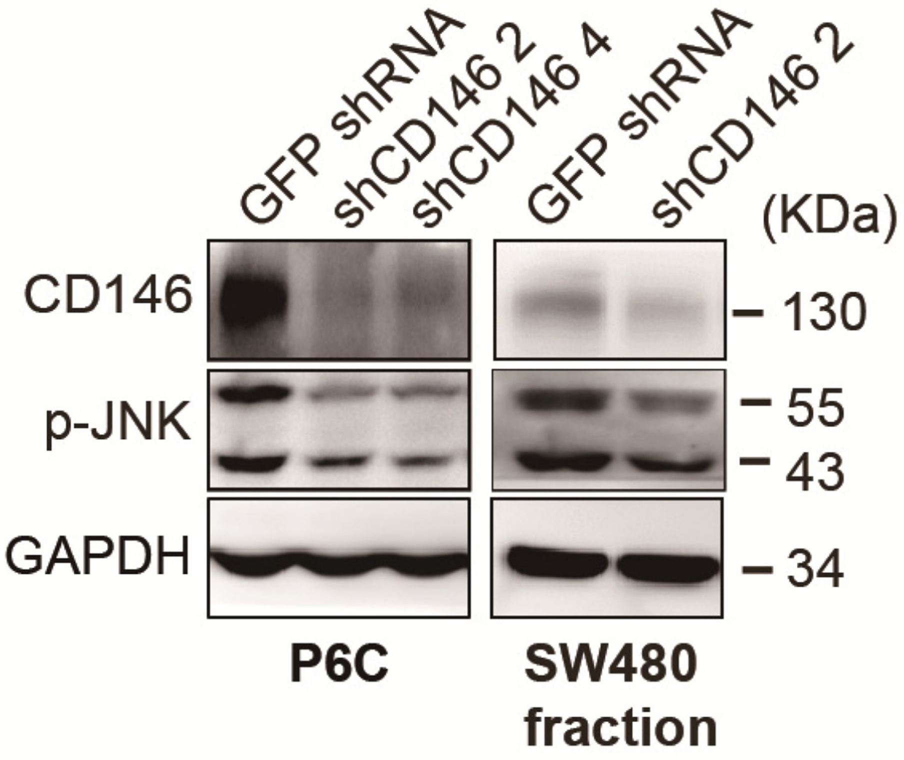

Supplementary Figure S8: Knockdown of CD146 inactivates non-canonical Wnt/ PCP pathway. JNK kinase activity was determined by western blotting in P6C cells and SW480 fraction.

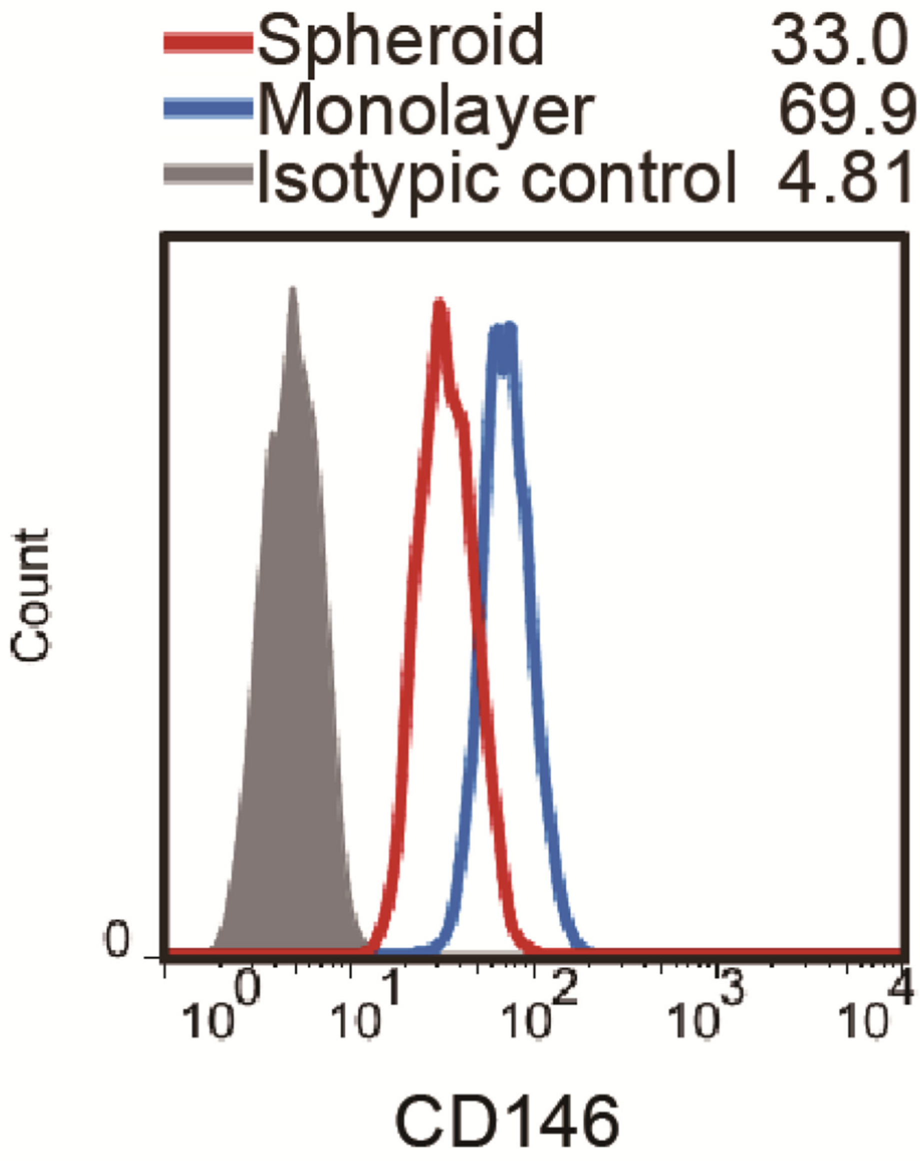

Supplementary Figure S9: CD146 is downregulated in spheres compared to the monolayer counterparts. P6C cells were cultured as adherent monolayer in medium containing 10% FBS. Spheroid cultures of P6C were maintained in ultra low adherent plates under SFM condition. Histograms of one representative experiment are shown.

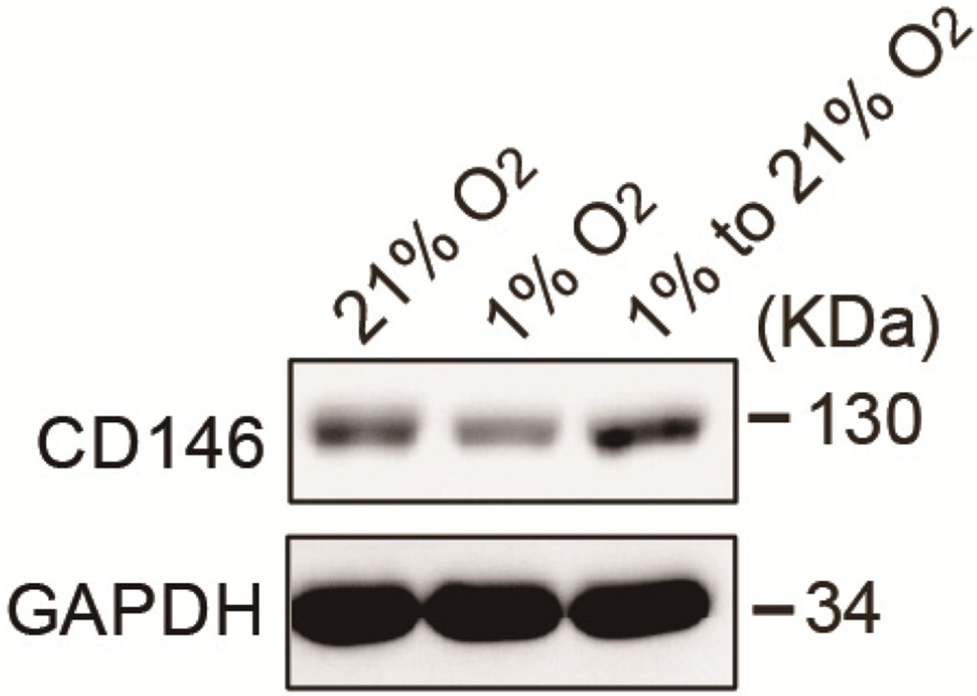

Supplementary Figure S10: CD146 is downregulated under hypoxic condition. P6C cells maintained in normoxic condition (21% O_2) were sub-cultured in hypoxia (1% O_2) for 24h and were then switched back to the normal condition.

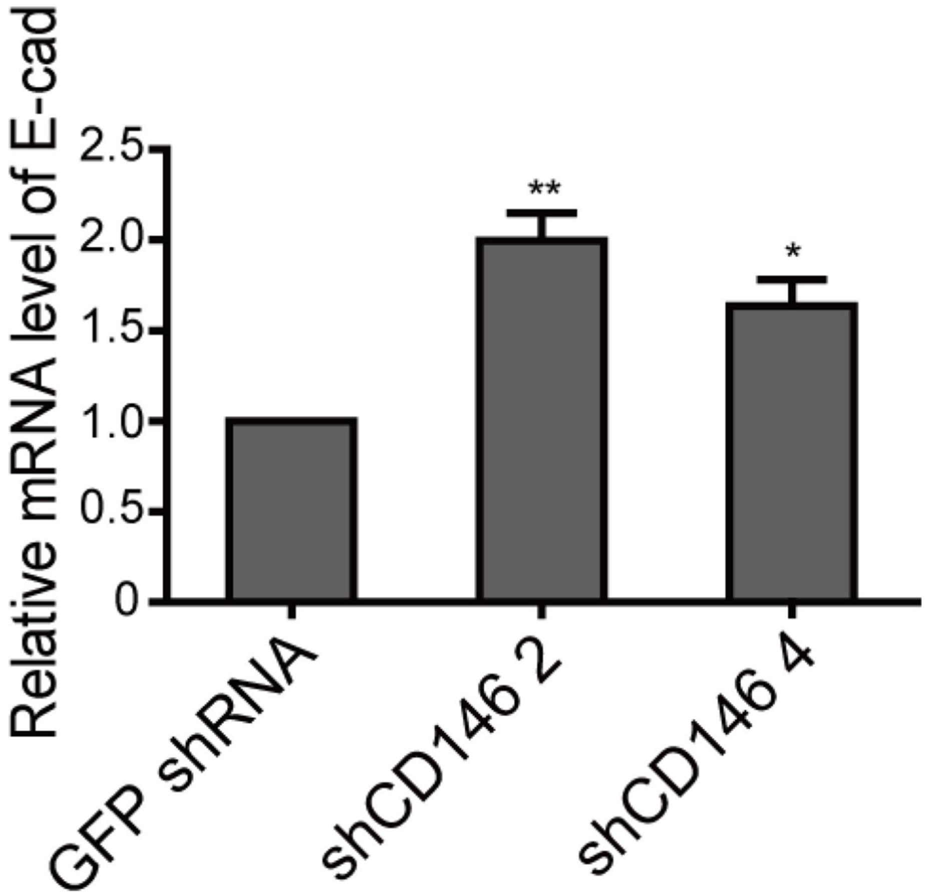

Supplementary Figure S11: Knockdown of CD146 induces the upregulation of E-cadherin in P6C cells. Relative mRNA level of E-cadherin was determined by qRT-PCR. **P<0.05, **P<0.01*. Error bars, mean ± s.d.

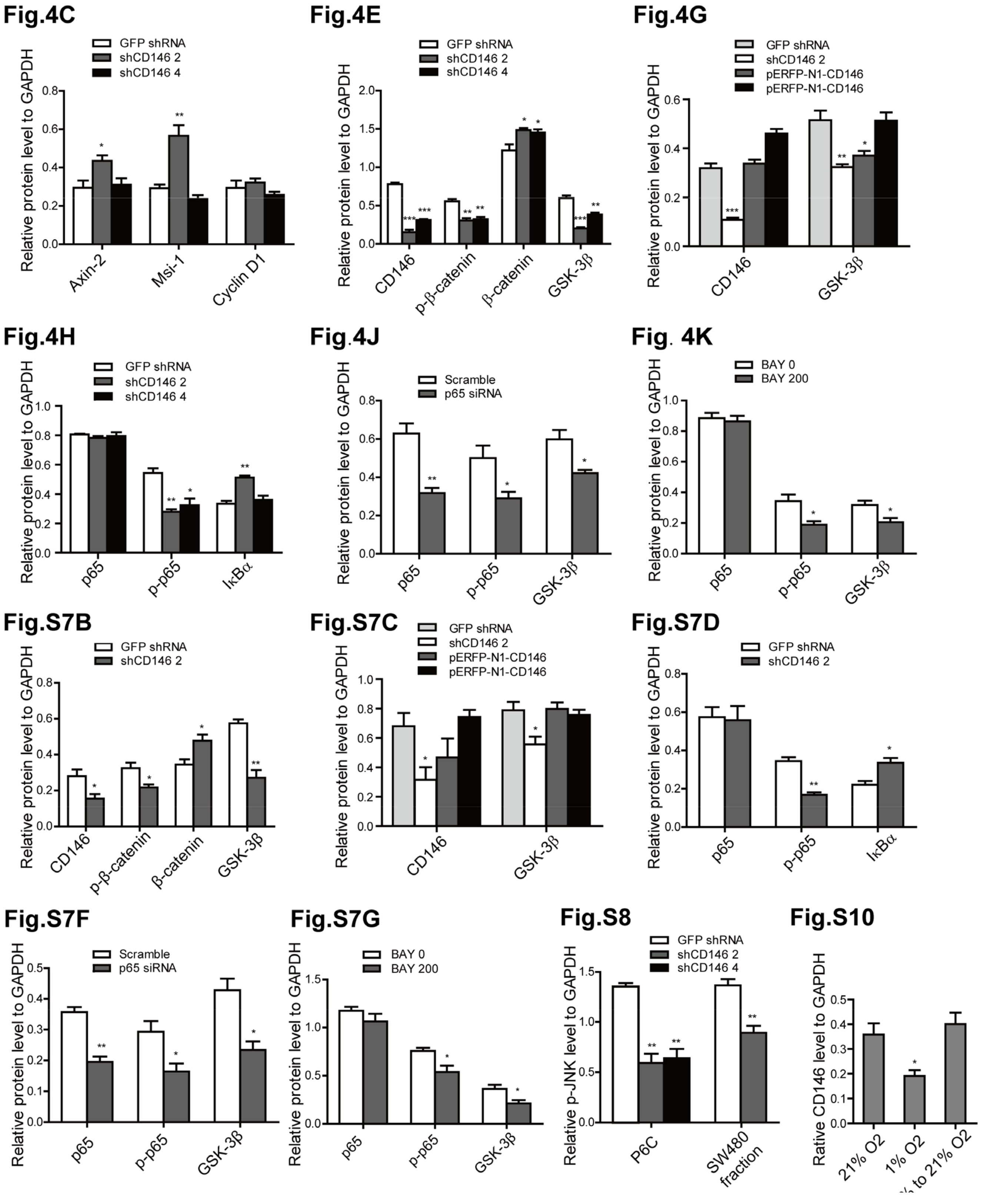

Supplementary Figure S12: Quantitative analysis of Western blot. Relative protein expression was determined by measuring the intensities of the bands and then normalizing to the internal control (GAPDH). **P<0.05,*P<0.01, ***P<0.001*. Error bars, mean ± s.d.

Supplementary Table S1: Pathway analysis based on the KEGG database

Pathway	Hits	Total	Percent	Enrichment test p-value	q-value
Wnt	20	151	13.25%	0.1627	0.7036
Notch	8	47	17.02%	0.1315	0.7036
Hedgehog	7	56	12.50%	0.3631	0.7055
ABC transporters	6	44	13.64%	0.314	0.7055

Hit threshold: fold change of gene expression≥2

Supplementary Table S2: Differentially expressed genes in P6C cells upon CD146 knockdown

Gene Symbol	Accession Number	Probe ID	Fold Change	
			shCD146 2 vs GFP shRNA	shCD146 4 vs GFP shRNA
LEF1	NM_001130714	221558_s_at	1.31	1.43
HNF1A	NM_002127	210515_at	1.63	1.51
CCND1	NM_053056	208712_at	1.35	0.81
AXIN2	NM_004655	222696_at	2.52	2.2
CD44	NM_000610	212063_at	1.14	1.36
BIRC5	NM_001012270	202094_at	0.93	0.66
ASCL2	NM_005170	207607_at	0.72	0.79
MSI1	NM_002442	206333_at	7.81	5.41
SOX9	NM_000346	202935_s_at	0.74	3.25
SOX2	NM_003106	213722_at	2.3	3.24
NANOG	NM_024865	220184_at	19.84	3.24
POU5F1	NM_002701	208286_x_at	1.7	0.78
WNT9A	NM_003395	230643_at	2.3	2.36
TWIST1	NM_000474	213943_at	5.66	1.35
EGFR	NM_005228	224999_at	0.97	0.89
FZD7	NM_003507	203705_s_at	3.01	1
EPCAM	NM_002354	201839_s_at	2.07	2.54
MYC	NM_002467	244089_at	1.05	1.98
NMYC	NM_005378	209757_s_at	23.09	47.22
JUN	NM_002228	201464_x_at	0.66	0.65
FGF9	NM_002010	206404_at	2.89	0.7
JAG1	NM_000214	209097_s_at	0.74	2.28
CDH1	NM_004360	201130_s_at	1.69	0.42
ABCB1	NM_000927	243951_at	2.63	1.77
RUNX2	NM_001015051	232231_at	1.32	4.78
CTNNB1	NM_001098209	201533_at	1.39	1.05
GSK3B	NM_001146156	209945_s_at	0.45	0.89
GSK3A	NM_019884	632_at	0.97	1.06
CREBBP	NM_001079846	228177_at	3.4	1.45
FZD1	NM_003505	204451_at	6.02	1.9
FZD3	NM_017412	227524_at	2.62	6.56
FZD8	NM_031866	224325_at	1.14	2.26
LRP5	NM_002335	209468_at	1.86	2.39
SFRP1	NM_003012	202035_s_at	0.48	0.32
DKK1	NM_012242	204602_at	0.45	0.98
MCAM	NM_006500	209087_x_at	0.14	0.23

Supplementary Table S3: shRNA used in CD146 knockdown

Name	Sequences	Respective cDNA locations
GFP shRNA	non-effective shRNA	nonsense sequence
shCD146 1	ATTCCTCAAGTCATCTGGT	506~524bp (190~196aa)
shCD146 2	GTTGAATCTGTCTTGTGAA	1299~1318bp (454~460aa)
shCD146 3	TGGCATTCAAGGAGAGGAA	1342~1360bp (468~474aa)
shCD146 4	GCTGGTTAAAGAAGACAAA	634~652bp(231~237aa)

Supplementary Table S4: Primers used in qRT-PCR

Gene	Sequences(5'-3')
MCAM (CD146)	Forward: TCAACGGCACGGCAAGTG<br>Reverse: AGGCCGTGCATTCAACACC
CD44s	TCATAGAAGGGCACGTGGTG<br>TGGGAGGTGTTGGATGTGAG
CD133	CTATTCAGGATATACTCTCAGCATT<br>TTTCTGTGGATGTAACTTTCAGTG
CD166	CGT CTG CTC TTC TGC CTC TT<br>TAG GTG CCT CAA ACA CGT TG
EpCAM	CGCAGCTCAGGAAGAATGTG<br>TGAAGTACACTGGCATTGACG
ITGB1 (Integrin β1)	TTCAGTGAATGGCAACAATG<br>AGCAACCACGCCTGCTAC
POU5F1 (Oct-4)	TTTAATCCCACATCATGTATCACT<br>CTATCTACTGTGTCCCAGG
SOX2	GTATCAGGAGTTGTCAAGGCAGAG<br>TCCTAGTCTTAAAGAGGCAGCAAAC
NANOG	CGATCTCCTGACCTTGT<br>CACGCCTGTAAATCCCA
MYC (c-Myc)	CGGAACTCTTGTGCGTAAGG<br>CTCAGCCAAGGTTGTGAGGT
BMI1	TTCGACCTTTGCAGATACCCATAAC<br>TGCCAATTGCTTCTAATGGAACAG
AXIN2	AGTGTGAGGTCCACGGAAAC<br>CTGGTGCAAAGACATAGCCA
MSI1	GGTTTCCAAGCCACAACCTA<br>TCGGGGAACTGGTAGGTGTA
CCND1 (Cyclin D1)	CTGGAGGTCTGCGAGGAACA<br>CCTTCATCTTAGAGGCCACGAA
HNF1A (TCF1)	AGGAGTGCAATAGGGCGGAATG<br>CCGGTTGGCAAACCAGTTGTAG
LEF1	CGAAGAGGAAGGCGATTTAG<br>CTGAGAGGTTTGTGCTTGTC
TWIST1	TGCCAATCAGCCACTGAAAG<br>TTTGCAGGCCAGTTTGATCCC
SOX9	TGGGCAAGCTCTGGAGACTTC<br>ATCCGGGTGGTCCTTCTTGTG
BIRC5 (Survivin)	AGAACTGGCCCTTCTTGGAGG<br>CTTTTTATGTTCCTCTATGGGGTC
WNT9A	GCAAGATGCTGGATGGGTC<br>GAGGATGGTCAGGGGCTC
E-cadherin	TGCCCAGAAAATGAAAAAGG<br>GTGTATGTGGCAATGCGTTC
GAPDH	CAGCCTCAAGATCATCAGCA<br>GTCTTCTGGTGGCAGTGAT

Supplementary Table S5: Primers used in ChIP assays

primers	Sequences(5’-3’)
Pair 1	TTCGCTTTCTTCTCCCCACC<br>GATTGGCTGGAAAACTCCGC
Pair 2	CGCTTTCTTCTCCCCACCTT<br>ATTGGCTGGAAAACTCCGCT
Pair 3	TCGCTTTCTTCTCCCCACCT<br>TTGGCTGGAAAACTCCGCT
